# Peri-Conceptional Folic Acid Supplementation and Children’s Physical Development: A Birth Cohort Study

**DOI:** 10.3390/nu15061423

**Published:** 2023-03-15

**Authors:** Shanshan Zhang, Mengting Yang, Xuemei Hao, Fu Zhang, Jixing Zhou, Fangbiao Tao, Kun Huang

**Affiliations:** 1 Department of Maternal, Child & Adolescent Health, School of Public Health, Anhui Medical University, Hefei 230032, China; 2 Key Laboratory of Population Health Across Life Cycle, Ministry of Education of the People’s Republic of China, Anhui Medical University, Hefei 230032, China; 3 NHC Key Laboratory of Study on Abnormal Gametes and Reproductive Tract, Hefei 230032, China; 4 Anhui Provincial Key Laboratory of Population Health and Aristogenics, Hefei 230032, China; 5 Scientific Research Center in Preventive Medicine, School of Public Health, Anhui Medical University, Hefei 230032, China

**Keywords:** folic acid, trajectory, adiposity rebound, birth cohort

## Abstract

Background: Maternal lack of folic acid supplementation during pregnancy may increase the risk of low birth weight and preterm delivery. However, little is known about the relationship between folic acid supplementation during pregnancy and the physical development of offspring in the later stage. Objective: This study aimed to explore the association between maternal folic acid supplementation status during pregnancy and the physical development of preschool children. Methods: A total of 3064 mother–child pairs with data on maternal folic acid supplementation status during pregnancy and children’s anthropometric measurements were recruited from the Ma’anshan-Anhui Birth Cohort (MABC) in China. Maternal folic acid supplementation status during pregnancy was the main exposure, and the primary outcomes were children’s growth development trajectories. Children’s growth development trajectories were fitted using group-based trajectory models. The association between maternal folic acid supplementation status during pregnancy and children’s growth trajectories was performed using multiple logistic regression models. Results: After adjusting for potential confounders, we found that the absence of maternal folic acid supplementation before pregnancy and in the first trimester was significantly associated with a “high level” trajectory (trajectory 3) and a “high rising level” trajectory (trajectory 4) of BMI-Z scores in children 0 to 6 years of age (OR = 1.423, 95%CI:1.022–1.982; OR = 1.654, 95%CI: 1.024–2.671). In children aged 4 to 6 years old, a “high level” trajectory (trajectory 3) of body fat ratio was substantially related to maternal no folic acid supplementation before pregnancy and in the first trimester (OR = 1.833, 95%CI:1.037–3.240). No significant additional benefits associated with physical developmental indicators in preschool children have been observed with continued folic acid supplementation after the first trimester of gestation. Conclusions: Maternal non-supplementation with folic acid during pregnancy is associated with a “high level” BMI trajectory and a “high level” body fat ratio trajectory in preschool-aged children.

## 1. Introduction

Folic acid, or vitamin B9, is one of the essential nutrients for human health. For pregnant women in particular, an adequate intake of folic acid is necessary for both maternal and children’s health. Peri-conceptional folic acid supplementation used for preventing fetal neural tube abnormalities has been well documented [1,2]. For this reason, the WHO recommends 400 µg per day during pregnancy (it should be started as early as possible (preferably before conception) to prevent neural tube defects). Some countries have implemented folic acid fortification strategies and advocated 400 µg per day of folic acid supplementation for pregnant women from the first month before pregnancy to the first trimester of pregnancy [3,4,5]. This evidence has also stimulated research into the association between folic acid supplementation during pregnancy and pregnancy outcomes. Increased intake of this vitamin reduces the risk of cleft lip and palate, pre-eclampsia, placental abruption, spontaneous abortion, and gestational hypertension [6,7,8,9,10]. Findings on the effect of folic acid supplementation during the peri-conceptional period (including preconception and the first trimester of pregnancy) on the offspring’s growth and development have been inconsistent. The Generation R Study reported that peri-conceptional folic acid supplementation resulted in higher birth weights and reduced the risk of low birth weight (LBW) and small for gestational age (SGA) [11]. The cohort study in two southern provinces of China found that maternal folic acid supplementation during pregnancy significantly reduces the risk of LBW and SGA [12]. In contrast, the Norwegian cohort study revealed no association between dietary folic acid intake, folic acid supplementation, or maternal plasma folic acid concentration measured in the second trimester of pregnancy and gestational age, infant birth weight, and head circumference [13].

The effect of peri-conceptional folic acid supplementation on the postnatal physical development of children is even less clear. The Generation R study found no association between maternal folic acid supplementation during pregnancy and infants’ head circumferences [14]. The Dutch KOALA birth cohort study indicated no association between maternal folic acid supplementation during pregnancy and children’s body mass index (BMI) at 1 year and 2 years of age after birth [15]. What is less clear is whether continued folic acid supplementation after the first trimester of pregnancy would bring long-term health benefits for children.

Therefore, we aimed to examine the relationship between peri-conceptional folic acid supplementation and preschool children’s BMI, head circumference, and body fat ratio (BF%) by using a large prospective birth cohort. We also attempted to identify the effect of continued folic acid supplementation after the first trimester of pregnancy on children’s physical development.

## 2. Methods

### 2.1. Study Population

This study was based on the Ma’anshan-Anhui Birth Cohort (MABC). From May 2013 to September 2014, pregnant women were consecutively recruited from antenatal clinics of the Maternal and Child Health Care Centre in Ma’anshan city in the Anhui Province of China. The Center contains about eighty percent of all pregnant women in Ma’anshan city every year. The inclusion criteria for women were set as: (1) within 14 gestational weeks; (2) planned to have antenatal checkups and childbirth in the Center; (3) could understand and complete the questionnaires; and (4) willing to participate in follow-ups during childhood. A total of 3474 eligible women were recruited. The study process was actually the natural course of pregnancy, childbirth, and children’s follow-up. Data on baseline information and nutritional supplementation of pregnant women during pregnancy, and the physical development of the children, have been collected prospectively. After excluding 39 twins and 162 adverse pregnancy outcomes, 3273 women with live singleton births were included. Women with missing data on folic acid supplementation in the first, second, or third trimester and one month before pregnancy were excluded. Children with BMI/head circumference/BF% measurements less than 3 times that of in the specified age range were further excluded.

The study was approved by the ethical committee of Anhui Medical University (20131401). Written informed consent was obtained from all participants.

### 2.2. Folic Acid Supplementation

Data on maternal folic acid supplementation were reported by women in questionnaires during the first, second, and third trimesters of pregnancy. 

Peri-conceptional folic acid supplementation was divided into 3 groups: (1) no supplementation in the first month before pregnancy and no supplementation in the first trimester of pregnancy; (2) supplementation either in the first month before pregnancy or in the first trimester of pregnancy; and (3) supplementation both before the first month of pregnancy and in the first trimester of pregnancy.

Folic acid supplementation after the first trimester of pregnancy was also divided into 3 groups: (1) routine supplementation in pre-pregnancy and in the 1st trimester of pregnancy; (2) continued supplementation in the 2nd or the 3rd trimesters of pregnancy; and (3) continued supplementation in both 2nd and 3rd trimesters of pregnancy.

The folic acid supplement dose was recommended by obstetricians as 400 µg per day.

### 2.3. Assessment of Children’s Physical Development

**Figure 1:**
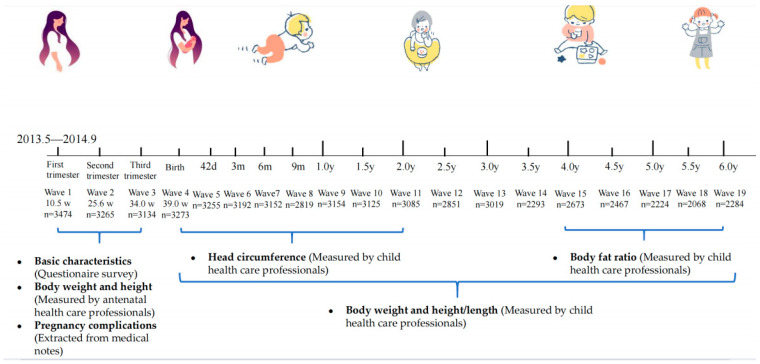
Timelines of cohort follow-up.

Children were followed up at 42 days after birth for the first time, then they were followed up every 3 months until 1 year of age, and children aged over 1 year were followed up after every six months until 6 years of age. Thus, a total of 15 follow-up visits were arranged for each child. Timelines of cohort follow-up and children’s developmental parameter measures are shown in Figure 1 and Table S1, respectively. Anthropometric measurements were performed by professional pediatric health practitioners including weight, length/height, and head circumference. Children’s head circumference was measured after two years of age, and the BF% was measured from four years of age.

From the obstetrical records kept during labor, birth weight, and length were extracted. A pan-style lever scale with a 0.01 kg accuracy was used to determine the infant’s weight. A sitting lever scale with a 0.05 kg accuracy was used to determine the toddler’s weight. A standard measuring bed accurate to 0.1 cm was used to gauge the infant’s length. Additionally, the children’s height and weight were determined using a mechanical child height and weight scale (Model: RRZ-50-RP) with an accuracy of 0.1 kg for weight and 0.1 cm for height. Children’s head circumference was measured with a soft ruler from the upper edge of the brow arch on the right side of the head, through the occipital ridge and the upper edge of the brow arch on the left side, and back to the starting point. The soft ruler was held close to the scalp and symmetrical to the left and right, to 1 decimal place in cm. 

BMI was calculated by dividing weight (kg) by the square of length/height (m^2^). The World Health Organization provides specific methods for standardizing children’s growth and development indicators on the website (https://www.who.int/tools/child-growth-standards/ accessed on 1 July 2022), and the method was run by using SPSS software. The relevant indicators mentioned above were converted to Z-scores for age and gender to obtain head circumference for age z-score (HC-Z) and BMI for age z-score (BMI-Z). 

Children were asked to take off their hats and shoes and to wear light clothing for body composition measurement. The InBody J20 body composition analyzer was applied to measure 15 electrical impedance measurements at 5 segmental sections (right upper limb, left upper limb, trunk, right lower limb, left lower limb) by means of 8-point contact electrodes. It is required that children keep stationary for 1 to 2 min. In this study, the body fat ratio was used as the key indicator of body composition.

### 2.4. Covariates

Confounding variables were identified by directed acyclic graph (DAG) [16] and included maternal age, maternal education years, place of residence, monthly household income per capita, parity, smoking/alcohol drinking during pregnancy, weight gain during pregnancy, gestational diabetes mellitus (GDM), and hypertensive disorders in pregnancy (HDP). 

Data on maternal age, education years, place of residence, family income, parity, and smoking/alcohol drinking during pregnancy were collected during the women’s first antenatal visit by questionnaire survey. In the first antenatal visit, women’s body weight and height were measured, and the weight was regarded as pre-pregnancy body weight. Gestational weight gain was calculated by weight recorded shortly before childbirth subtracting the weight before pregnancy. Information on GDM and HDP was abstracted from the obstetrical notes.

Meanwhile, data on birth weight by gestational age, children’s sex, exclusive breastfeeding within 6 months, and diet during childhood were included in the sensitivity analyses. Information on gestational age, birth weight, and children’s sex was extracted from medical records. Information on exclusive breastfeeding for the first 6 months and diet during childhood was reported by the children’s caregivers in the follow-up questionnaire survey.

## 3. Statistical Analysis

### 3.1. Trajectory Modeling

**Figure 2:**
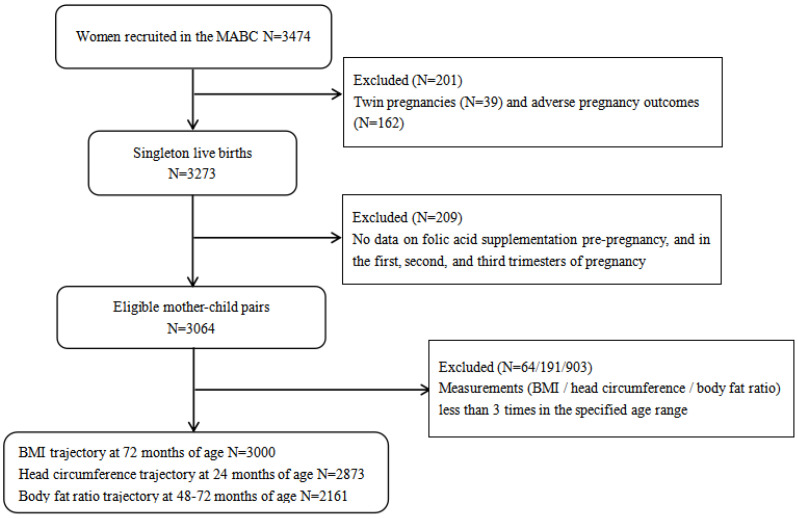
Flowchart of study participants.

Sample selection: Typically, trajectory fitting requires a minimum of three measurements. This is because fewer measurements would limit the modeling capabilities and, therefore, the type and number of trajectories that can be generated [17,18,19]. To ensure the quality of the trajectory fit, 64 children with BMI data less than 3 times before 6 years old were excluded, as well as 191 children with head circumference measurement less than 3 times within 2 years of age and 903 children with body composition assessment less than 3 times in 4–6 years of age (Figure 2).

Group-based Trajectory: A group-based trajectory model (GBTM) was employed to find different trends in the development of children from birth to age six. The GBTM is a latent class growth model created to find groups of trajectories or groups of people who have similar trajectories throughout time. The Bayesian information criteria were used in GBTM to establish the ideal number of trajectory groups with various trajectory patterns (BIC). In general, the groups with the greatest BIC and polynomial values are picked in that order. Typically, modeling can be conducted using objective criteria, but subjective judgment is also important [20]. The professional expertise can support the polynomial order or the number of groups. The final model should accurately reflect the properties of the data while remaining straightforward [20]. High-order terms (four-order terms) were employed to fit the trajectories due to the numerous age groups included and the significant temporal variability of BMI in the early stages of child development. Based on the model’s parameters, the best model was chosen. According to the BIC of each fitted model, the number of categories used for category fitting grew from two until an acceptable trajectory category and a suitable combination of higher terms were chosen.

In our study, the HC-Z and BF% trajectories were identified as three-category trajectories (the parameters were statistically significant and the maximum BIC values were −16,998.6 and −25,569.8, respectively). The BMI-Z trajectory had the maximum BIC value (−41,934.7) when fitting to the four categories, and the parameters were significant.

For the assessment of the children’s adiposity rebound (AR), we fit a fractional polynomial mixed-effects model and refer to the article by Wen. The detailed analysis method has been described in our published articles [21,22]. In the present study, age at AR before 60 months was defined as early AR (EAR). According to the estimated AR for each child, therefore, children were divided into EAR and non-EAR.

### 3.2. Association between Folic Acid Supplementation Status and Children’s Growth Trajectories and AR

IBM SPSS Statistics 23.0 was used to analyze the relationship between folic acid supplementation and children’s physical developmental trajectories. Multiple logistic regression models were used for the relationship between folic acid supplementation and children’s trajectories on BMI- Z scores, HC-Z scores, and BF% with trajectory two as the reference. The independent variables were referenced in the group with supplementation both before the first month of pregnancy and in the first trimester of pregnancy. For the association between folic acid supplementation status and AR, non-early AR was regarded as the reference in the dependent variable. After the first trimester of pregnancy, independent variables were referenced in the group with routine supplementation in pre-pregnancy and in the first trimester of pregnancy. A *p*-value of less than 0.05 was considered statistically significant.

We conducted two sensitivity analyses for folic acid routine supplementation and continued supplementation for both. First, folic acid supplementation might be a protective factor for preterm birth and low birth weight. In turn, preterm birth and low birth weight were associated with physical development in children [11,12,13,20]. Thus, gestational week and birth weight may be potential mediators of the association between maternal folic acid supplementation and children’s physical development and were further adjusted. In the second sensitivity analysis, exclusive breastfeeding and children’s diet were reported to be strongly associated with their physical development [23]. As these variables were not regarded as relating to maternal folic acid supplementation, they were served as the precision variables and were further adjusted to improve the accuracy.

## 4. Results

The basic characteristics of the included participants are shown in Table 1. The average age of pregnant women was 26.6 years, and the average length of education was 13.4 years. Nulliparous accounted for 88.9%. Most mothers never smoked (95.8%) or drank alcohol (92.0%) during pregnancy, with GDM and HCP accounting for 13% and 6%, respectively.

### 4.1. Trajectory Fitting of Physical Development Indicators

The BMI-Z trajectories were divided into four groups: trajectory one was the “low level” trajectory group (16.7%), trajectory two was the “middle level” trajectory group (40%), trajectory three was the “high level” trajectory group (32.8%), and trajectory four was the “higher rising level” trajectory group (10.5%).

The HC-Z trajectories were classified into three groups, i.e., trajectory one was the “low level” trajectory group, accounting for 29.4%; trajectory two was the “middle level” trajectory group, accounting for 51.1%; and trajectory three was the “high rising level” trajectory group, accounting for 19.5%.

**Figure 3:**
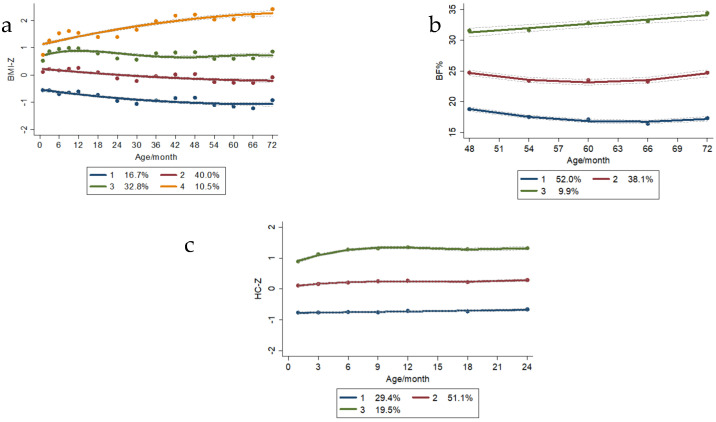
Children’s physical development trajectory. (**a**) BMI−z trajectory; (**b**) Body fat ratio trajectory; (**c**) HC−Z trajectory.

The BF% trajectories were categorized into three groups: trajectory one was the “low level” trajectory group (52.0%), trajectory two was the “middle level” trajectory group (38.1%), and trajectory three was the “high rising level” trajectory, accounting for 9.9% (Figure 3).

### 4.2. Association between Peri-Conceptional Folic Acid Supplementation and Child Growth and Development Indicators

As shown in Table 2, Table 3, Table 4 and Table 5, it was found that no supplementation of folic acid before pregnancy and in the first trimester was significantly associated with a “high level” trajectory (trajectory 3) (OR = 1.423, 95%CI: 1.022–1.982) and a “high rising level” trajectory (trajectory 4) (OR = 1.654, 95%CI: 1.024–2.671) of children’s BMI-Z scores. Similarly, we found that no supplementation of folic acid before pregnancy and in the first trimester was significantly associated with a “high level” trajectory (trajectory 3) of BF% in children of 4 to 6 years of age (OR = 1.833, 95%CI: 1.037–3.240). No significant association was found between peri-conceptional folic acid supplementation status and children’s head circumference development or children’s EAR. Sensitivity analysis did not change the main analysis results (Tables S6–S9).

### 4.3. Association between Continued Folic Acid Supplementation after the First Trimester of Pregnancy and Children’s Growth and Development

No significant additional benefits associated with physical developmental indicators in preschool children were observed with continued folic acid supplementation after the first trimester of pregnancy, i.e., in the second and third trimesters of pregnancy (Tables S2–S5). Sensitivity analysis also did not change the results (Tables S10–S13).

## 5. Discussion

In the present study, we found that no maternal supplementation of folic acid before pregnancy and in the first trimester was associated with high-level BMI and body fat trajectories. No additional benefit for children’s physical development was observed from continued folic acid supplementation after 12 weeks of gestation.

Previous studies have focused on the effect of maternal folic acid supplementation on children’s birth outcomes, mainly reflecting the intrauterine growth and development of the fetus [11,12,13]. The association between maternal folic acid supplementation and early offspring’s BMI development was not observed in the previous cohort, possibly due to the small number of follow-up time points and the lack of observations on later BMI development. BMI fluctuates in the early stage of life [24]. BMI at a single time point may not be able to reflect the dynamic growth and development of children. BMI trajectories at multiple time points, however, will better reflect the potential tendency of an individual’s physical development in a certain course of life. Studies have confirmed that a stable high BMI trajectory in childhood is associated with an increased risk of high BMI, overweight, and obesity in the later stage [25,26]. Meanwhile, the body fat ratio reflects the proportion of adipose tissue in the body more accurately, which is the main contributor to obesity-related health outcomes. It also optimizes the traditional strategy of isolated measurements of BMI in illustrating its potential health impact. Our findings also provide evidence that early childhood body fat development may be influenced by peri-conceptional folic acid supplementation.

Maternal nutrition in early pregnancy can significantly influence the intrauterine environment, which may also greatly affect the long-term development of the offspring [27,28], especially during the critical period of fetal development. An experimental analysis of hypertensive rats showed that folate deficiency promoted a variety of phenotypes, such as oxidative stress and metabolic syndrome [29]. Studies have shown that folic acid supplementation may improve insulin resistance, another aggravating feature of obesity [30,31].

At present, the exact mechanism explaining the effect of maternal folic acid supplementation during pregnancy on children’s physical development is not clear. However, folic acid consumption of one carbon unit participates in the folic acid cycle, thus regulating DNA and RNA metabolism, as well as amino acid metabolism, and affecting fetal growth and development [32]. To some extent, it may involve a key role in children’s metabolism.

Regarding the continuation of folic acid supplementation after the third trimester, our study did not currently find any additional benefit to a child’s physical development. However, a randomized controlled trial study in Northern Ireland found that continued folic acid supplementation after 3 months of pregnancy could benefit neurocognitive development in children [33]. Animal studies have confirmed that excessive folic acid supplementation increases DNA CG methylation, which alters epigenetic mechanisms, suppresses immune gene expression, and ultimately promotes asthma development [34]. An Australian study reported that folic acid supplementation in late pregnancy was associated with an increased risk of asthma at 3.5 years of age and an increased risk of persistent asthma under 5 years of age [35]. Haberg et al. conducted a case-control study and concluded that elevated levels of folic acid during pregnancy were associated with an increased risk of asthma in children at 3 years of age [36].

Our study has several strengths. To our knowledge, it is the most extensive study examining peri-conceptional folic acid supplementation and the offspring’s longest physical development. Children were followed up every 3 months after birth and every 6 months after 1 year of age. To date, we have collected complete data on children at 15 follow-up visits. The frequent follow-ups will allow for an accurate fit to the children’s growth trajectory. Furthermore, based on a prospective birth cohort, information on exposures, outcomes, and confounders can be accurately collected and thus reduce recall bias.

There are also some limitations in this study. Information on maternal diet during pregnancy was not collected to consider the folic acid supplementation in the diet. The actual dose of folic acid supplementation for pregnant women is not known, although it is as prescribed by the doctor (400 µg per day). This may somewhat confound the grouping of exposure. An assay of internal exposure to folic acid may better reflect the actual intake. Secondly, paternal genetic factors may play an essential role in influencing children’s physical development. For example, paternal overweight and obesity and their potential inheritance are risk factors for children’s high BMI [37]. Anthropometric indicators of fathers are still lacking in the current study. In addition, we collected maternal pre-pregnancy BMI and weight gain during pregnancy, but other indicators of the maternal constitution, such as pelvic inlet, body composition, and placental size were not collected, and these factors might affect children’s physical development [38]. Finally, we only observed children up to 6 years of age. Their development in late childhood and the prevalence of overweight and obesity in later life require a longer follow-up.

In conclusion, maternal non-supplementation with folic acid during pregnancy may be associated with high-level trajectories of BMI and body fat ratio trajectory in preschool-aged children.

## Figures and Tables

**Table 1:** Basic characteristics of participants (*n* = 3064).

Variables	Total
Demographic Characteristics	
Maternal age (years, mean ± SD)	26.6 ± 3.6
Education level (years, mean ± SD)	13.4 ± 3.1
Place of residence [*n* (%)]	
Urban areas	2810 (91.7)
Rural areas	254 (8.3)
Monthly household income per capita [yuan, *n* (%)]	
<2500	807 (26.3)
2500–4000	1319 (43.0)
>4000	938 (30.6)
Maternal characteristics	
Smoking during pregnancy [*n* (%)]	
Yes	129 (4.2)
No	2935 (95.8)
Alcohol drinking during pregnancy [*n* (%)]	
Yes	245 (8.0)
No	2819 (92.0)
Weight gain during pregnancy * (kg) (Mean ± SD) (*n* = 3058)	17.9 ± 5.1
Parity [*n* (%)]	
Primiparity	2725 (88.9)
Multiparity	339 (11.1)
GDM [*n* (%)]	
Yes	398 (13.0)
No	2666 (87.0)
HDCP [n (%)] * (*n* = 3057)	
Yes	182 (6.0)
No	2875 (93.8)

* indicating variables with missing values: 6 in weight gain during pregnancy and 7 in HDCP.

**Table 2:** Association of peri-conceptional folic acid supplementation status with BMI-Z trajectory in children 0–6 years of age [n = 3000, OR (95%CI)].

Folic Acid Supplementation Status	Model 1			Model 2		
	Traj 1	Traj 3	Traj 4	Traj 1	Traj 3	Traj 4
No supplementation both in pre-pregnancy and in the 1st trimester of pregnancy	1.122 (0.748–1.685)	1.434 (1.041–1.975) *	1.719 (1.088–2.715) *	1.43 (0.750–1.743)	1.423 (1.022–1.982) *	1.654 (1.024–2.671) *
Supplementation in pre-pregnancy or in the 1st trimester of pregnancy	0.901 (0.722–1.124)	0.963 (0.804–1.153)	1.233 (0.939–1.619)	0.910 (0.724–1.142)	0.954 (0.793–1.149)	1.224 (0.923–1.623)

For folic acid supplementation status, supplementation both in pre-pregnancy and in the 1st trimester of pregnancy was the reference group. For BMI trajectories, Traj 2 was regarded as the reference group. When observing the impact of folic acid supplementation status exposure in the first trimester of pregnancy, folic acid supplementation status in the second and third trimesters of pregnancy was further adjusted. Model 1: Crude model. Model 2: Adjusted for maternal age, education level, place of residence, monthly household income per capita, smoking, alcohol drinking, weight gain during pregnancy, parity, GDM, and HCP. *: *p* < 0.05.

**Table 3:** Association of peri-conceptional folic acid supplementation status with body fat ratio trajectory in children 4–6 years of age [n = 2161, OR (95%CI)].

Folic Acid Supplementation Status	Model 1		Model 2	
	Traj 1	Traj 3	Traj 1	Traj 3
No supplementation both in pre-pregnancy and in the 1st trimester of pregnancy	1.418 (0.994–2.023)	1.802 (1.041–3.117)	1.379 (0.954–1.995)	1.833 (1.037–3.240)
Supplementation in pre-pregnancy or in the 1st trimester of pregnancy	1.066 (0.881–1.289)	1.109 (0.797–1.542)	1.086 (0.892–1.323)	1.151 (0.820–1.616)

For folic acid supplementation status, supplementation both in pre-pregnancy and in the 1st trimester of pregnancy was the reference group. For body fat ratio trajectories, Traj 2 was regarded as the reference group. When observing the impact of folic acid supplementation status exposure in the first trimester of pregnancy, folic acid supplementation status in the second and third trimesters of pregnancy was further adjusted. Model 1: Crude model. Model 2: Adjusted for maternal age, education level, place of residence, monthly household income per capita, smoking, alcohol drinking, weight gain during pregnancy, parity, GDM, and HCP.

**Table 4:** Association of peri-conceptional folic acid supplementation status with head circumference-z-score trajectory in children 0–2 years of age [n = 2873, OR (95%CI)].

Folic Acid Supplementation Status	Model 1		Model 2	
	Traj 1	Traj 3	Traj 1	Traj 3
No supplementation both in pre-pregnancy and in the 1st trimester of pregnancy	1.031 (0.747–1.423)	0.858 (0.586–1.254)	1.024 (0.734–1.429)	1.004 (0.677–1.487)
Supplementation in pre-pregnancy or in the 1st trimester of pregnancy	1.059 (0.884–1.270)	0.942 (0.766–1.157)	1.048 (0.870–1.263)	0.982 (0.794–1.215)

For folic acid supplementation status, supplementation both in pre-pregnancy and in the 1st trimester of pregnancy was the reference group. For head circumference trajectories, Traj 2 was regarded as the reference group. When observing the impact of folic acid supplementation status exposure in the first trimester of pregnancy, folic acid supplementation status in the second and third trimesters of pregnancy was further adjusted. Model 1: Crude model. Model 2: Adjusted for maternal age, education level, place of residence, monthly household income per capita, smoking, alcohol drinking, weight gain during pregnancy, parity, GDM, and HCP.

**Table 5:** Association of peri-conceptional folic acid supplementation status with children’s early adiposity rebound [n = 2267, OR (95%CI)].

Folic Acid Supplementation Status	Model 1	Model 2
No supplementation both in pre-pregnancy and in the 1st trimester of pregnancy	1.175 (0.855–1.614)	1.110 (0.798–1.544)
Supplementation in pre-pregnancy or in the 1st trimester of pregnancy	1.048 (0.879–1.249)	1.004 (0.837–1.203)

For folic acid supplementation status, supplementation both in pre-pregnancy and in the 1st trimester of pregnancy was the reference group. For AR, non-EAR was regarded as the reference group. When observing the impact of folic acid supplementation status exposure in the first trimester of pregnancy, folic acid supplementation status in the second and third trimesters of pregnancy was further adjusted. Model 1: Crude model. Model 2: Adjusted for maternal age, education level, place of residence, monthly household income per capita, smoking, alcohol drinking, weight gain during pregnancy, parity, GDM, and HCP.

## Data Availability

The datasets generated and/or analyzed during the current study are not publicly available but are available from the corresponding author at reasonable request.

## Supplementary Materials

The following supporting information can be downloaded at: https://www.mdpi.com/article/10.3390/nu15061423/s1, Table S1. Children’s developmental parameters at different ages [Mean (SD)]. Table S2. Association of continued periconceptional folic acid supplementation with BMI-Z trajectory in children 0–6 years [n = 1084, OR (95%CI)]. Table S3. Association of continued periconceptional folic acid supplementation status with body fat ratio trajectory in children 4–6 years of age [n = 792, OR (95%CI)]. Table S4. Association of continued periconceptional folic acid supplementation status with head circumference-z-score trajectory in children 0–2 years of age [n = 1054, OR (95%CI)]. Table S5. Association of continued periconceptional folic acid supplementation status with children’s early adiposity rebound [n = 832, OR (95%CI)]. Table S6. Association of periconceptional folic acid supplementation status with BMI-Z trajectory in children 0–6 years of age [n = 3000, OR (95%CI)]. Table S7. Association of periconceptional folic acid supplementation status with body fat ratio trajectory in children 4–6 years of age [n = 2161, OR (95%CI)]. Table S8. Association of periconceptional folic acid supplementation status with head circumference-z-score trajectory in children 0–2 years of age [n = 2873, OR (95%CI)]. Table S9. Association of periconceptional folic acid supplementation status with children’s early adiposity rebound [n = 2267, OR (95%CI)]. Table S10. Association of continued periconceptional folic acid supplementation status with BMI-Z trajectory in children 0–6 years of age [n = 1084, OR (95%CI)]. Table S11. Association of continued periconceptional folic acid supplementation status with body fat ratio trajectory in children 4–6 years of age [n = 792, OR (95%CI]. Table S12. Association of continued periconceptional folic acid supplementation status with head circumference-z-score trajectory in children 0–2 years of age [n = 1054, OR (95%CI)]. Table S13. Association of continued periconceptional folic acid supplementation status with children’s early adiposity rebound [n = 832, OR (95%CI)].
