# Supplementary material for: Peri-Conceptional Folic Acid Supplementation and Children’s Physical Development: A Birth Cohort Study
Source: Nutrients. 2023 Mar 15;15(6):1423. doi: 10.3390/nu15061423 (PMC10052637; doi:10.3390/nu15061423)
Supplement: Supplementary file 1 [file nutrients-15-01423-s001.zip › nutrients-2250321-supplementary.pdf]

Table S1. Children's developmental parameters at different ages [Mean (SD)]

	BMI-z	BF%	HC-Z
Birth	-0.01 (1.01)	-	-0.09 (1.13)
42 days	0.20 (0.89)	-	0.01 (0.84)
3 months	0.40 (0.96)	-	0.08 (0.83)
6 months	0.42 (1.04)	-	0.14 (0.86)
9 months	0.48 (0.98)	-	0.16 (0.87)
1 year	0.49 (0.93)	-	0.19 (0.86)
1.5 years	0.32 (0.87)	-	0.16 (0.86)
2 years	0.13 (0.90)	-	0.21 (0.86)
2.5 years	0.08 (0.99)	-	-
3 years	0.29 (1.02)	-	-
3.5 years	0.39 (1.07)	-	-
4 years	0.39 (1.11)	22.29 (6.12)	-
4.5 years	0.13 (1.09)	21.21 (6.24)	-
5 years	0.10 (1.11)	21.11 (6.34)	-
5.5 years	0.09 (1.20)	20.68 (6.42)	-
6 years	0.35 (1.25)	21.78 (6.95)	-

Continued supplementation

Table S2. Association of continued periconceptional folic acid supplementation with BMI-Z trajectory in children 0-6 years[n=1084, OR (95%CI)]

Folic acid supplementation status	Model 1			Model 2		
	Traj 1	Traj 3	Traj 4	Traj 1	Traj 3	Traj 4
**Continued supplementation in the 2nd or the 3rd trimesters of pregnancy**	0.816 (0.494-1.350)	1.221 (0.838-1.779)	0.933 (0.499-1.779)	0.825 (0.495-1.377)	1.269 (0.864-1.862)	1.009 (0.533-1.908)
**Continued Supplementation supplementation in both 2nd and 3rd trimesters of pregnancy**	1.878 (1.011-3.486)	1.580 (0.908-2.751)	1.656 (0.743-3.692)	1.810 (0.955-3.429)	1.578 (0.892-2.793)	1.671 (0.731-3.816)

For folic acid supplementation status, routine supplementation in pre-pregnancy and in the 1sttrimester of pregnancy was the reference group. For BMI trajectories, Traj 2 was regarded as the reference group.

Model 1: Crude model.

Model 2: Adjusted for maternal age, education level, Place of residence, monthly household income per capita, smoking, alcohol drinking, weight gain during pregnancy, parity, GDM and HCP.

Table S3. Association of continued periconceptional folic acid supplementation status with body fat ratio trajectory in children 4-6 years of age[n=792, OR (95%CI]

Folic acid supplementation status	Model 1		Model 2	
	Traj 1	Traj 3	Traj 1	Traj 3
**Continued supplementation in the 2nd or the 3rd trimesters of pregnancy**	1.222 (0.801-1.863)	0.989 (0.469-2.088)	1.286 (0.835-1.983)	0.993 (0.464-2.126)
**Continued Supplementation supplementation in both 2nd and 3rd trimesters of pregnancy**	0.640 (0.378-1.085)	0.367 (0.109-1.235)	0.586 (0.340-1.011)	0.368 (0.107-1.266)

For folic acid supplementation status, routine supplementation in pre-pregnancy and in the 1sttrimester of pregnancy was the reference group. For body fat ratio trajectories, Traj2 was regarded as the reference group.

Model 1: Crude model.

Model 2: Adjusted for maternal age, education level, Place of residence, monthly household income per capita, smoking, alcohol drinking, weight gain during pregnancy, parity, GDM and HCP;

Table S4. Association of continued periconceptional folic acid supplementation status with head circumference-z-score trajectory in children 0-2 years of age[n=1054, OR (95%CI)]

Folic acid supplementation status	Model 1		Model 2	
	Traj 1	Traj 3	Traj 1	Traj 3
**Continued supplementation in the 2nd or the 3rd trimesters of pregnancy**	1.436 (0.982-2.099)	1.239 (0.798-1.924)	**1.506 (1.021-2.222)**	1.255 (0.802-1.964)
**Continued Supplementation supplementation in both 2nd and 3rd trimesters of pregnancy**	0.887 (0.503-1.565)	1.378 (0.790-2.404)	0.866 (0.482-1.556)	1.299 (0.730-2.310)

For folic acid supplementation status, routine supplementation in pre-pregnancy and in the 1sttrimester of pregnancy was the reference group. For head circumference trajectories, Traj2 was regarded as the reference group.

Model 1: Crude model.

Model 2: Adjusted for maternal age, education level, Place of residence, monthly household income per capita, smoking, alcohol drinking, weight gain during pregnancy, parity, GDM and HCP;

Table S5. Association of continued periconceptional folic acid supplementation status with children's early adiposity rebound [n=832, OR (95%CI)]

Folic acid supplementation status	Model 1	Model 2
**Continued supplementation in the 2nd or the 3rd trimesters of pregnancy**	0.957 (0.653-1.403)	0.953 (0.645-1.410)
**Continued Supplementation supplementation in both 2nd and 3rd trimesters of pregnancy**	0.705 (0423-1.177)	0.696 (0.411-1.181)

For folic acid supplementation status, routine supplementation in pre-pregnancy and in the 1sttrimester of pregnancy was the reference group. For AR, Not EAR was regarded as the reference group.

Model 1: Crude model.

Model 2: Adjusted for maternal age, education level, Place of residence, monthly household income per capita, smoking, alcohol drinking, weight gain during pregnancy, parity, GDM and HCP;

sensitivity analyses

pre-pregnancy and in the 1st trimester of pregnancy

Table S6. Association of periconceptional folic acid supplementation status with BMI-Z trajectory in children 0-6 years of age[n=3000, OR (95%CI)]

Folic acid supplementation status	Model 3			Model 4		
	Traj 1	Traj 3	Traj 4	Traj 1	Traj 3	Traj 4
**No Supplementation both in pre-pregnancy and in the 1st trimester of pregnancy**	1.155 (0.756-1.766)	**1.433 (1.027-1.999)**	**1.652 (1.016-2.684)**	1.143 (0.746-1.752)	**1.467 (1.049-2.051)**	**1.702 (1.048-2.765)**
**Supplementation in pre-pregnancy or in the 1st trimester of pregnancy**	0.920 (0.731-1.157)	0.958 (0.795-1.154)	1.219 (0.916-1.622)	0.908 (0.722-1.144)	0.942 (0.781-1.137)	1.236 (0.927-1.647)

For folic acid supplementation status, Supplementation both in pre-pregnancy and in the 1st trimester of pregnancy was the reference group. For BMI trajectories, Traj 2 was regarded as the reference group.

Model 3 Further adjusted for birth weight by gestational age+children's sex

Model 4 Further adjusted for exclusive breastfeeding for the first 6 months + diet during childhood

Table S7. Association of periconceptional folic acid supplementation status with body fat ratio trajectory in children 4-6 years of age[n=2161, OR (95%CI)]

Folic acid supplementation status	Model 3		Model 4	
	Traj 1	Traj 3	Traj 1	Traj 3
**No Supplementation both in pre-pregnancy and in the 1st trimester of pregnancy**	1.383 (0.956-2.002)	**1.833 (1.037-3.242)**	1.347 (0.927-1.958)	**1.867 (1.051-3.316)**
**Supplementation in pre-pregnancy or in the 1st trimester of pregnancy**	1.090 (0.895-1.328)	1.151 (0.820-1.616)	1.080 (0.885-1.318)	1.146 (0.811-1.619)

For folic acid supplementation status, Supplementation both in pre-pregnancy and in the 1st trimester of pregnancy was the reference group. For body fat ratio trajectories, Traj 2 was regarded as the reference group.

Model 3 Further adjusted for birth weight by gestational age+children's sex

Model 4 Further adjusted for exclusive breastfeeding for the first 6 months + diet during childhood

Table S8. Association of periconceptional folic acid supplementation status with head circumference-z-score trajectory in children 0-2 years of age[n=2873, OR (95%CI)]

Folic acid supplementation status	Model 3		Model 4	
	Traj 1	Traj 3	Traj 1	Traj 3
**No Supplementation both in pre-pregnancy and in the 1st trimester of pregnancy**	1.063 (0.758-1.490)	1.001 (0.672-1.490)	1.022 (0.730-1.431)	1.002 (0.676-1.487)
**Supplementation in pre-pregnancy or in the 1st trimester of pregnancy**	1.075 (0.890-1.299)	0.972 (0.783-1.206)	1.047 (0.867-1.263)	0.971 (0.783-1.203)

For folic acid supplementation status, Supplementation both in pre-pregnancy and in the 1st trimester of pregnancy was the reference group. For head circumference trajectories, Traj 2 was regarded as the reference group.

Model 3 Further adjusted for birth weight by gestational age+children's sex

Model 4 Further adjusted for exclusive breastfeeding for the first 6 months + diet during childhood

Table S9. Association of periconceptional folic acid supplementation status with children's early adiposity rebound [n=2267, OR (95%CI)]

Folic acid supplementation status	Model 3	Model 4
**No Supplementation both in pre-pregnancy and in the 1st trimester of pregnancy**	1.105(0.794-1.539)	1.161(0.795-1.697)
**Supplementation in pre-pregnancy or in the 1st trimester of pregnancy**	0.999(0.832-1.198)	1.005(0.816-1.238)

For folic acid supplementation status, supplementation both in pre-pregnancy and in the 1st trimester of pregnancy was the reference group. For AR, Not EAR was regarded as the reference group.

Model 3 Further adjusted for birth weight by gestational age+children's sex

Model 4 Further adjusted for exclusive breastfeeding for the first 6 months + diet during childhood

Continued supplementation

Table S10. Association of continued periconceptional folic acid supplementation status with BMI-Z trajectory in children 0-6 years of age[n=1084, OR (95%CI)]

Folic acid supplementation status	Model 3			Model 4		
	Traj 1	Traj 3	Traj 4	Traj 1	Traj 3	Traj 4
Continued supplementation in the 2nd or the 3rd trimesters of pregnancy	0.815 (0.486-1.367)	1.257 (0.854-1.850)	0.989 (0.519-1.884)	0.851 (0.464-1.560)	1.103 (0.703-1.731)	0.616 (0.275-1.381)
Continued Supplementation supplementation in both 2nd and 3rd trimesters of pregnancy	1.836 (0.961-3.507)	1.520 (0.853-2.707)	1.529 (0.654-3.575)	1.996 (0.984-4.047)	1.273 (0.663-2.442)	1.035 (0.389-2.754)

For folic acid supplementation status, routine supplementation in pre-pregnancy and in the 1sttrimester of pregnancy was the reference group. For BMI trajectories, Traj 2 was regarded as the reference group.
Model 3 Further adjusted for birth weight by gestational age+children's sex
Model 4 Further adjusted for exclusive breastfeeding for the first 6 months + diet during childhood

Table S11. Association of continued periconceptional folic acid supplementation status with body fat ratio trajectory in children 4-6 years of age[n=792, OR (95%CI]

Folic acid supplementation status	Model 3		Model 4	
	Traj 1	Traj 3	Traj 1	Traj 3
Continued supplementation in the 2nd or the 3rd trimesters of pregnancy	1.297 (0.841-2.001)	0.999 (0.465-2.146)	1.230 (0.760-1.991)	0.901 (0.382-2.124)
Continued Supplementation supplementation in both 2nd and 3rd trimesters of pregnancy	0.592 (0.342-1.024)	0.400 (0.116-1.382)	0.543 (0.292-1.011)	0.429 (0.120-1.532)

For folic acid supplementation status, routine supplementation in pre-pregnancy and in the 1sttrimester of pregnancy was the reference group. For body fat ratio trajectories, Traj2 was regarded as the reference group.
Model 3 Further adjusted for birth weight by gestational age+children's sex
Model 4 Further adjusted for exclusive breastfeeding for the first 6 months + diet during childhood

Table S12. Association of continued periconceptional folic acid supplementation status with head circumference-z-score trajectory in children 0-2 years of age[n=1054, OR (95%CI)]

Folic acid supplementation status	Model 3		Model 4	
	Traj 1	Traj 3	Traj 1	Traj 3
**Continued supplementation in the 2nd or the 3rd trimesters of pregnancy**	1.530 (1.033-2.265)	1.217 (0.771-1.921)	1.519 (1.027-2.245)	1.263 (0.806-1.979)
**Continued Supplementation supplementation in both 2nd and 3rd trimesters of pregnancy**	0.856 (0.473-1.550)	1.258 (0.696-2.272)	0.902 (0.502-1.622)	1.313 (0.737-2.340)

For folic acid supplementation status, routine supplementation in pre-pregnancy and in the 1sttrimester of pregnancy was the reference group. For head circumference trajectories, Traj2 was regarded as the reference group.

Model 3 Further adjusted for birth weight by gestational age+children's sex

Model 4 Further adjusted for exclusive breastfeeding for the first 6 months + diet during childhood

Table S13. Association of continued periconceptional folic acid supplementation status with children's early adiposity rebound [n=832, OR (95%CI)]

Folic acid supplementation status	Model 3	Model 4
**Continued supplementation in the 2nd or the 3rd trimesters of pregnancy**	0.955 (0.645-1.414)	0.957 (0.645-1.419)
**Continued Supplementation supplementation in both 2nd and 3rd trimesters of pregnancy**	0.724 (0.425-1.231)	0.717 (0.422-1.218)

For folic acid supplementation status, routine supplementation in pre-pregnancy and in the 1sttrimester of pregnancy was the reference group. For AR, Not EAR was regarded as the reference group.

Model 3 Further adjusted for birth weight by gestational age+children's sex

Model 4 Further adjusted for exclusive breastfeeding for the first 6 months + diet during childhood
